# Inactivation gating of Kv7.1 channels does not involve concerted cooperative subunit interactions

**DOI:** 10.1080/19336950.2018.1441649

**Published:** 2018-03-01

**Authors:** Eshcar Meisel, William Tobelaim, Meidan Dvir, Yoni Haitin, Asher Peretz, Bernard Attali

**Affiliations:** Department of Physiology & Pharmacology, the Sackler Faculty of Medicine and Sagol School of Neurosciences, Tel Aviv University, Tel Aviv, Israel

**Keywords:** Kv7, KCNQ, cooperativity, inactivation, potassium channel

## Abstract

Inactivation is an intrinsic property of numerous voltage-gated K^+^ (Kv) channels and can occur by N-type or/and C-type mechanisms. N-type inactivation is a fast, voltage independent process, coupled to activation, with each inactivation particle of a tetrameric channel acting independently. In N-type inactivation, a single inactivation particle is necessary and sufficient to occlude the pore. C-type inactivation is a slower process, involving the outermost region of the pore and is mediated by a concerted, highly cooperative interaction between all four subunits. Inactivation of Kv7.1 channels does not exhibit the hallmarks of N- and C-type inactivation. Inactivation of WT Kv7.1 channels can be revealed by hooked tail currents that reflects the recovery from a fast and voltage-independent inactivation process. However, several Kv7.1 mutants such as the pore mutant L273F generate an additional voltage-dependent slow inactivation. The subunit interactions during this slow inactivation gating remain unexplored. The goal of the present study was to study the nature of subunit interactions along Kv7.1 inactivation gating, using concatenated tetrameric Kv7.1 channel and introducing sequentially into each of the four subunits the slow inactivating pore mutation L273F. Incorporating an incremental number of inactivating mutant subunits did not affect the inactivation kinetics but slowed down the recovery kinetics from inactivation. Results indicate that Kv7.1 inactivation gating is not compatible with a concerted cooperative process. Instead, adding an inactivating subunit L273F into the Kv7.1 tetramer incrementally stabilizes the inactivated state, which suggests that like for activation gating, Kv7.1 slow inactivation gating is not a concerted process.

## Introduction

Inactivation is an inherent property of many voltage-gated K^+^ channels (Kv). Inactivation of Kv channels can occur by N-type or/and C-type mechanisms. The fast N-type inactivation involves an intracellular peptide domain at the N-terminus of α or β subunits which occludes the channel central cavity and prevents ion permeation [1–3]. The slow C-type inactivation was suggested to involve structural rearrangements in the outer pore leading to a loss of K^+^ coordination sites in the selectivity filter [4–7]. The biophysical hallmarks of C-type inactivation are reflected by its inhibition by high external K^+^ or external tetraethylammonium (TEA) [1,8,9]. These features have been interpreted as a ‘foot in the door’ mechanism, in which occupancy of an ion binding site by K^+^ or TEA at the external filter entrance slows or prevents the conformational changes required for C-type inactivation [5]. Along this line, in *Shaker* K^+^ channels, a Ba^2+^ binding site was identified topologically below the C-type inactivation gate [10].

The Kv7.1 channel (or KCNQ1) is the only member out of five of a subfamily of voltage-gated K^+^ channels, Kv7, which undergoes inactivation. The five Kv7 (Kv7.1–5) channel members play important functions in various tissues including brain, heart, kidney, stomach, pancreas or inner ear [11]. Kv7.1 α subunits can interact with each of the five KCNE β subunits, displaying distinct current characteristics [12,13]. Co-assembly of Kv7.1 with KCNE1 produces the *I_KS_* current, which together with IKr (hERG channel) form the main repolarizing currents of the cardiac action potential [14–16]. Mutations in either Kv7.1 or KCNE1 genes lead to life-threatening cardiac arrhythmias causing long or short QT syndromes (LQT or SQT) and atrial fibrillation [17,18]. Similar to all Kv channels, the Kv7.1 structure features six transmembrane segments (S1-S6) containing a voltage-sensing module (S1–S4) and a pore domain (S5–S6). In contrast to *Shaker*-like Kv channels, Kv7.1 does not harbor an N-terminal T1 tetramerization domain, but possesses a large C-terminus, which was shown to be important for channel gating, assembly and trafficking [19–23]. The Kv7.1 C-terminus comprises amphipathic α helices that form three coiled-coils. The proximal helices A and B, adjacent to the membrane, form a coiled-coil that binds calmodulin (CaM) [24–27], whereas the distal helices C and D form tandem coiled-coils that serve as a tetramerization domain [21,23]. A recent cryo-EM study showed that each Kv7.1 subunit associates with one calmodulin molecule [28]. Unique structural features within the S4-S5 linker permit uncoupling of the voltage sensor from the pore in the absence of PIP2 [28].

*Shaker* channels have provided a powerful tool to establish the activation gating mechanisms of voltage-dependent K^+^ channels, implying prior independent movement of all four voltage sensor domains (VSD), followed by channel opening via a last concerted cooperative transition. Using voltage-clamp fluorometry, it was shown that in Kv7.1 channels the four S4s move relatively independently and that the more S4s have moved to their activated state, the higher the probability of transition from closed to open channel [29,30]. Similarly, using a thermodynamic mutant cycle analysis and a concatenated tetrameric Kv7.1 channel, we showed a sequential activation gating for Kv7.1 with weakly cooperative gating transitions, suggesting an incremental contribution of each subunit to channel opening even before all VSDs have activated [31]. What about the inactivation gating of Kv7.1 channels? Is inactivation gating of Kv7.1 concerted or not? We previously characterized the Kv7.1 LQT pore S5 mutant L273F, which exhibits a voltage-dependent slow inactivation [32]. We found that this slow inactivation delays entry of Ba^2+^ ions to the pore and traps them by slowing their exit from the selectivity filter [32]. To determine the nature of subunit interactions along Kv7.1 slow inactivation gating, we used a concatenated tetrameric Kv7.1 channel and introduced into one, two, three and four subunits the slow inactivating pore mutation L273F. Inserting an incremental number of inactivating mutant subunits into the Kv7.1 tetramer did not affect the inactivation kinetics but slowed down the recovery time from inactivation. There is also a progressive increase in the extent of slow inactivation, which stabilizes the inactivated state of the Kv7.1 tetrameric channel and suggests that like activation, inactivation gating is not a concerted process.

## Results

### Concatenated tetrameric L273F Kv7.1 mutant channels display similar properties to those of the monomeric L273F Kv7.1 in CHO cells

**Figure 1.**
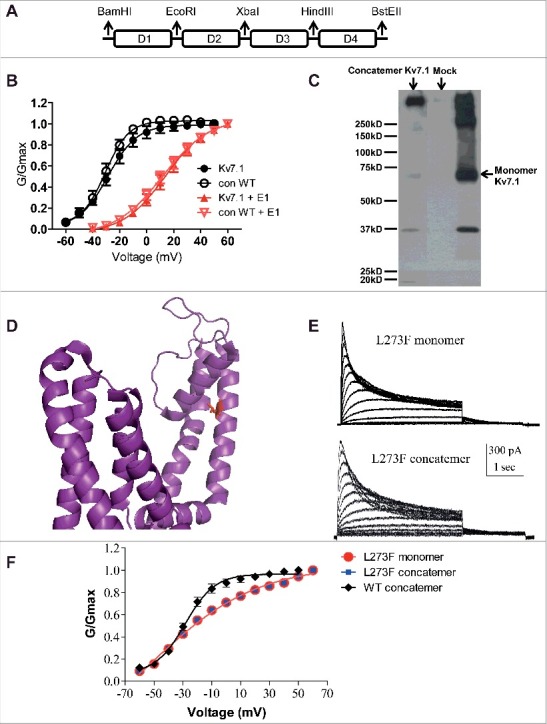
Characterization of the WT and L273F mutant Kv7.1 concatenated tetrameric channels. A, Top panel, scheme of the Kv7.1 concatenated tetrameric channel construct (Con), where subunits D1, D2, D3 and D4 are connected by flexible linkers. Each linker (8 glycines) harbors a unique restriction site. B, conductance-voltage relations of WT monomeric and WT concatenated tetrameric Kv7.1 constructs in the absence or presence of KCNE1. Data were fitted with a single Boltzmann function. C, Western blot showing lysates from HEK293 transfected with empty vector (Mock), KCNQ1 monomeric and concatenated tetrameric constructs. D, location of residue L273 in the S5 α helical segment of the Kv7.1 pore domain as mapped in the recent cryo-EM structure of the frog Kv7.1 (Sun, 2017). E, representative current traces of L273F Kv7.1 monomeric and concatenated tetrameric mutant constructs. CHO cells were held at −90 mV and the membrane voltage was stepped for 3 s from −60 mV to +60 mV in 10 mV increments and then repolarized for 1.5 s to −60 mV. F, conductance-voltage relations of the WT Kv7.1 concatemer, L273F Kv7.1 monomer and L273F Kv7.1 concatemer. Data were fitted with a single Boltzmann function.

As described previously [31], the WT Kv7.1 concatenated tetrameric construct (Con) was first built into the pGEM vector, where D_1_, D_2_, D_3_, and D_4_ subunits were connected by flexible linkers (8 glycines), each harboring unique restriction sites (Fig. 1A). WT Con was subsequently used as a template for constructing the L273F inactivating mutant subunit combinations. WT Con was then inserted into the pcDNA3 vector to allow eukaryotic expression in CHO and HEK 293 cells. This strategy enabled us to cut and paste mutated subunits in any chosen combination. Concatenated tetrameric channel constructs were previously used for examining homo- and heterotetrameric assembly, cooperativity in intersubunit interactions, and inactivation [33–38].

To confirm that our construct was expressed as a concatenated tetrameric channel protein, we compared its expression in HEK 293 cells with that of the monomeric Kv7.1 protein by examining its molecular mass in SDS-PAGE under reducing conditions followed by Western blotting. While the monomeric Kv7.1 construct appeared as a main immunoreactive band of about 70 kDa accompanied by higher molecular mass aggregates, the concatenated construct expressed a major high molecular mass immunoreactive band of more than 280 kDa, roughly corresponding to the tetrameric channel protein (Fig. 1C). At the functional level, the electrophysiological properties of the WT and S5 pore mutant L273F monomeric constructs and their concatenated tetrameric Kv7.1 counterparts were similar (Fig. 1B, D, E and F). Figure 1B shows that in the absence and presence of KCNE1, the WT Con exhibited similar voltage dependence to that of the WT monomeric Kv7.1 construct (WT monomeric Kv7.1, V_50_ = −28.6 ± 0.5 mV; s = 10.8 ± 0.4 mV, n = 6; WT Con, V_50_ = −30.6 ± 0.6 mV; s = 8.7 ± 0.5 mV, n = 6; WT monomeric Kv7.1+KCNE1, V_50_ = 13.9 ± 0.6 mV; s = 15.6 ± 0.7 mV, n = 10; WT Con Kv7.1+KCNE1, V_50_ = 11.5 ± 0.9 mV; s = 17.4 ± 1.0 mV, n = 12). Figure 1D shows the location of residue L273 in the S5 α helical segment of the Kv7.1 pore domain as mapped in the recent cryo-EM structure of the frog Kv7.1 [28]. Figure 1E and 1F indicate that the tetrameric concatemer L273F mutant displayed similar inactivation kinetics and voltage dependence to that of the L273F monomeric Kv7.1 construct (L273F monomeric Kv7.1, V_50_ = −24.0 ± 2.5 mV; s = 27.2 ± 3.4 mV, n = 12; L273F Con, V_50_ = −22.2 ± 2.3 mV; s = 26.9 ± 2.7 mV, n = 10). Interestingly, the L273F slow inactivating mutant, either in its monomeric or in its concatenated configuration displayed a significant right-shift in the voltage dependence of activation and an increase in the Boltzmann slope compared to WT Kv7.1 channels (For WT Con and L273F Con, ΔV_50_ = +8.4 mV and Δs = +18.2 mV).

### Insertion of an incremental number of inactivating L273F mutant subunits does not affect the inactivation kinetics but progressively increases the fractional inactivation of the Kv7.1 tetrameric channel

**Figure 2.**
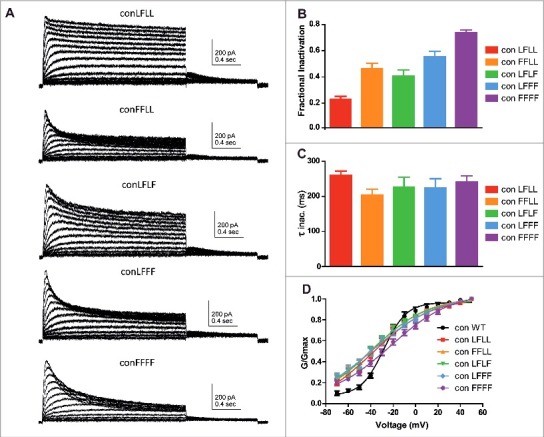
Expression of concatenated Kv7.1 subunits bearing an increasing number of L273F mutations. A, representative current traces of the following constructs: ConD_2_L273F (ConLFLL), ConD_2,4_L273F (ConLFLF), ConD_1,2_L273F (ConFFLL), ConD_,2,3,4_L273F (ConLFFF) and ConD_1,2,3,4_L273F (ConFFFF). CHO cells were held at −90 mV and the membrane voltage was stepped for 2 s from −70 mV to +50 mV in 10 mV increments and then repolarized for 1 s to −60 mV. B, Fractional inactivation F of the different concatemer constructs was measured at +50 mV (n = 7–10). C, Time constants of inactivation relaxation measured at +50 mV and determined by one exponential fit of the inactivation traces (n = 8–14). D, conductance-voltage relations of the different concatemer constructs. Data were fitted with a single Boltzmann function (n = 7–10).

To examine the nature of subunit interactions along Kv7.1 inactivation gating, the L273F inactivating mutation was progressively introduced into the concatenated tetrameric Kv7.1 channel to form the following constructs: ConD_2_L273F (ConLFLL), ConD_1,2_L273F (ConFFLL), ConD_2,4_L273F (ConLFLF), ConD_,2,3,4_L273F (ConLFFF) and ConD_1,2,3,4_L273F (ConFFFF). Figure 2A shows that following the incremental incorporation of the L273F mutation into the subunits of the concatenated tetrameric channel, there is a progressive increase in the extent of slow inactivation. The increasing number of incorporated L273F mutant subunits enhances the fractional inactivation. When measured at +50 mV, the fractional inactivation (see methods) became increasingly larger with an incremental number of subunits bearing the L273F mutation, with 0.22 ± 0.02 (n = 7) for ConLFLL, 0.45 ± 0.4 (n = 10) for ConFFLL, 0.39 ± 0.04 (n = 8) for ConLFLF, 0.54 ± 0.04 (n = 8) for ConLFFF and 0.73 ± 0.02 (n = 10) for ConFFFF (Fig. 2B). Interestingly, the incremental number of mutant subunits incorporated into the Kv7.1 channel tetramer did not significantly affect the time constants of the inactivation kinetics (Fig. 2C; one way ANOVA, P = 0.5679, F = 0.7413; ConLFLL, τ_inact_ = 260 ± 13 ms, n = 8; ConFFLL, τ_inact_ = 204 ± 16 ms, n = 13; ConLFLF, τ_inact_ = 227 ± 28 ms, n = 12; ConLFFF, τ_inact_ = 224 ± 27 ms, n = 14; ConFFFF, τ_inact_ = 241 ± 18 ms, n = 14). Although the Boltzmann slopes of the various L273F mutant combinations were shallower compared to WT Con, no significant difference in their voltage dependence was found, except for a right-shift trend observed with ConFFFF as noted above (Fig. 2D; ConLFLL, V_50_ = – 38.1 ± 6.4 mV, n = 7; ConFFLL, V_50_ = −38.6 ± 3.5 mV, n = 10; ConLFLF, V_50_ = −38.4 ± 5.2 mV, n = 8; ConLFFF, V_50_ = −37.6 ± 3.4 mV, n = 8; ConFFFF, V_50_ = −22.2 ± 2.3 mV, n = 10).

### Inserting an incremental number of inactivating mutant subunits increases the slope of the voltage-dependence of steady-state inactivation and slows down the recovery time from inactivation

**Figure 3.**
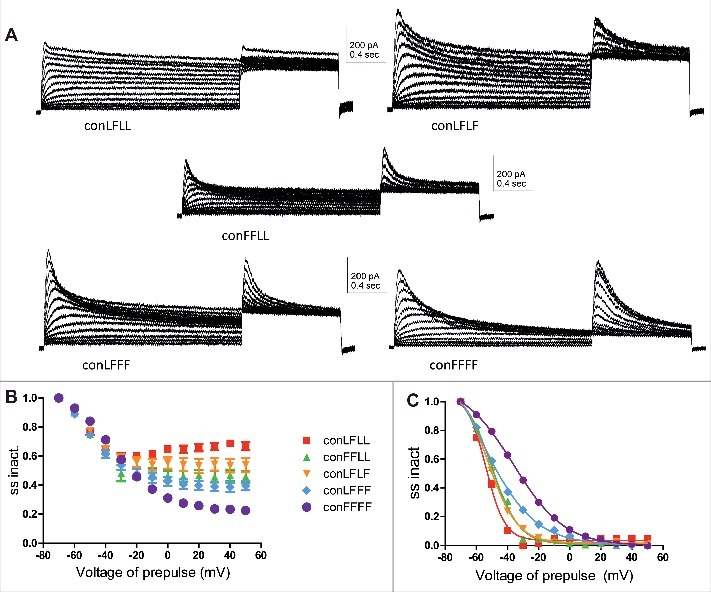
Steady-state inactivation of the different concatemer constructs. A, representative steady-state inactivation current traces of the different concatemer constructs, where the membrane potential was stepped to varying pre-pulse voltages from −70 mV to +50 mV in increments of 10 mV and to a subsequent test pulse of +50 mV. B, The different peak currents measured at the test pulse at +50 mV were divided by the largest peak current (at +50 mV) obtained from the −70 mV prepulse. These ratios of steady-state inactivation were plotted against the prepulse voltage (n = 7–10). C, The normalized ratios of steady-state inactivation were plotted against the prepulse voltage Data were fitted with a single Boltzmann function.

**Figure 4.**
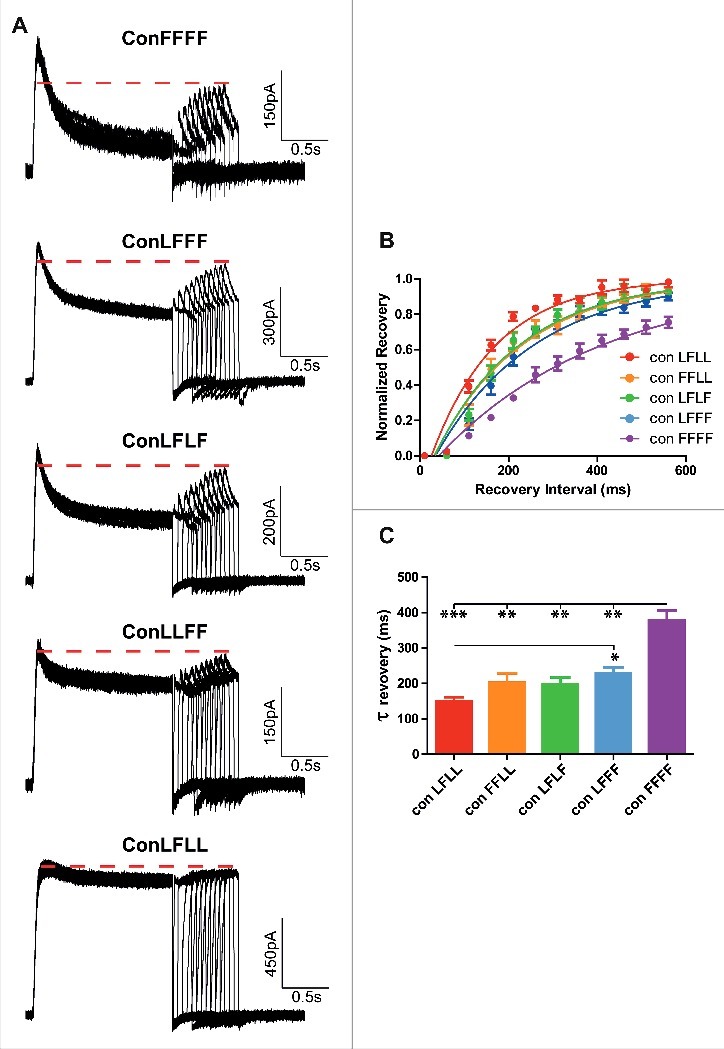
Recovery from inactivation of the different concatemer constructs. A, representative current traces of the recovery from inactivation of the different concatemer constructs. The recovery time from inactivation was determined from a −90 mV holding potential, by stepping the membrane voltage for 1.5 s to +50 mV to open the various Kv7.1 concatemer channels (first pulse), then repolarizing to −90 mV for various times to allow recovery from inactivation and stepping back to +50 mV for 0.5 s to reopen the channels (second pulse). B, The fractional recovery was calculated by the peak current of the second pulse divided by the peak of the first pulse. These ratios were plotted against the recovery times (n = 4). C, time constants of the recovery time from inactivation were calculated from an exponential fit (one way ANOVA, n = 4; *P < 0.01, **P < 0.001, ***P < 0.0001).

To determine the steady-state inactivation of each L273F construct, the membrane potential was stepped to varying pre-pulse voltages from −70 mV to +50 mV in increments of 10 mV and to a subsequent test pulse at +50 mV (Fig 3). The different peak currents measured at the test pulse at +50 mV were divided by the largest peak current (at +50 mV) obtained from the −70 mV prepulse. These ratios of steady-state inactivation were plotted against the prepulse voltage (Fig. 3B) and subsequently normalized (Fig. 3C). With an incremental number of subunits bearing the L273F mutation, we observed a gradual increase in the slope of the voltage-dependence of steady-state inactivation, accompanied a progressive increase in the extent of inactivation and a right-shift observed only for ConFFFF (Fig. 3C: ConLFLL, V_50_ = −53.8 ± 1.1 mV, s = −6.1 ± 0.8 mV, n = 7; ConFFLL, V_50_ = −50.2 ± 1.4 mV, s = −8.8 ± 1.0 mV, n = 10; ConLFLF, V_50_ = −52.4 ± 0.8 mV, s = −9.3 ± 0.5 mV, n = 8; ConLFFF, V_50_ = −57.2 ± 2.7 mV, s = −16.8 ± 1.1 mV, n = 8; ConFFFF, V_50_ = −36.2 ± 0.4 mV, s = −16.3 ± 0.3 mV, n = 10). The recovery time from inactivation was determined from a −90 mV holding potential, by stepping the membrane voltage for 1.5 s to +50 mV to open the various Kv7.1 concatemer channels (first pulse), then repolarizing to −90 mV for various times to allow recovery from inactivation and stepping back to +50 mV for 0.5 s to reopen the channels (second pulse) (Fig. 4A). The fractional recovery was calculated by the peak current of the second pulse divided by the peak of the first pulse. These ratios were plotted against the recovery times and the time constants of recovery from inactivation were calculated from an exponential fit (Fig. 4B). Results show that inserting an incremental number of inactivating mutant subunits increases the recovery time from inactivation (Fig. 4B and C: one way ANOVA, *P* < 0.0001, F = 15.43; ConLFLL τ_recov_ = 150 ± 11 ms, n = 4; ConFFLL, τ_recov_ = 203 ± 26 ms, n = 4; ConLFLF, τ_recov_ = 199 ± 18 ms, n = 4; ConLFFF, τ_recov_ = 228 ± 17 ms, n = 7; ConFFFF, τ_recov_ = 379 ± 27 ms, n = 4; *P < 0.01, **P < 0.001, ***P < 0.0001).

## Discussion

The purpose of the present study was to explore the nature of subunit interactions along Kv7.1 slow inactivation gating and to address the following questions. Is a conformational change in one Kv7.1 subunit sufficient for slow macroscopic inactivation to happen, or does it occur with the four subunits in a concerted fashion? Alternatively, does Kv7.1 slow inactivation imply a graded contribution of each subunit via non-concerted conformational changes? For that purpose, we used concatenated tetrameric Kv7.1 channels and introduced sequentially the slow inactivating pore mutation L273F into the four subunits.

Two different mechanisms, also known as N-type and C-type inactivation have been described to cause the inactivation of Kv channels. N-type inactivation is rather well understood and has been shown to occur through a “ball-and-chain” mechanism [1, 2] as originally proposed for Na^+^ channel inactivation [39,40]. N-type inactivation is voltage independent, coupled to activation, and fast, being complete within a few milliseconds in *Shaker* B channels. A single inactivation particle is necessary and sufficient for N-type inactivation to occlude the pore and prevent ion conduction, with each inactivation particle of a tetrameric channel acting independently [3,41]. In contrast, the mechanism of C-type inactivation is not fully understood yet. C-type inactivation of Kv1 channels [42] has been attributed to a constriction of the external region of the selectivity filter [43,44], and is mediated by highly cooperative subunit interactions [37,45,46]. However, it was recently proposed that C-type inactivation does not imply a pore constriction [47] and instead, might result from a subtle pore dilation at the outermost region of the selectivity filter [48]. In Kv1 channels, C-type inactivation is voltage independent and very slow, while in hERG1 channels (Kv11.1) it is fast and voltage-dependent. C-type inactivation of hERG1 K^+^ channels is mediated by a concerted, highly cooperative interaction between all four subunits [49].

Inactivation of Kv7.1 channels does not exhibit the hallmarks of N- and C-type inactivation. Inactivation of WT Kv7.1 channels is invisible macroscopically but can be revealed by hooked tail currents, which reflects the recovery from a fast and voltage-independent inactivation process [50,51]. The fast voltage-independent flicker possibly reflects small-scale fluctuating motions of the selectivity filter [50,51]. A recent study [57] showed that the Kv7.1 channel opens when the voltage sensor activates to both an intermediate and an activated state, resulting in intermediate (IO) and activated (AO) channel open states. The AO state was found to be coupled to the activated state of the VSD, while the IO state is coupled to the intermediate state of the VSD, via different mechanisms, with the VSD-pore coupling being less efficient in opening the pore in the AO state. Thus, a fraction of Kv7.1 channels may show upon depolarization a smaller total current during the transitions from the IO to AO states, giving rise to the “hooked tail current” inactivation phenotype following repolarization [57]. Remarkably, Kv7.1 channels exhibit an additional voltage-dependent slow inactivated state to which a minor fraction of the WT Kv7.1 channel population can transit but to which a large portion of several Kv7.1 mutants can populate [52–55]. We previously showed that the Kv7.1 pore mutant L273F displays a large voltage-dependent inactivation process with slow relaxation and recovery kinetics, distinct from the C-type inactivation observed in *Shaker* channels [32]. We hypothesized that the voltage-dependent slow inactivation of Kv7.1 mutant L273F arises from increased rigidity of the carbonyl ring of Tyr^315^ at the upper filter and a stronger coordination of K^+^ at filter site S_1_^32^. This slow inactivation was found to delay entry of Ba^2+^ ions to the pore and trap them by slowing their exit from the selectivity filter. Furthermore, external potassium ions were found to accelerate inactivation kinetics and exacerbate barium trapping. This feature contrasts to C-type inactivation of *shake*r potassium channel, which is inhibited by elevated potassium concentrations [1,9,42,56]. In line with our previous assumption of an additional slow voltage-dependent inactivated state, the recovery from inactivation of L273F channels was recently shown to exhibit two components, a fast phase that is similar to that observed in the hooked tail currents of WT Kv7.1, and an additional slow phase that reflects the recovery from a slow inactivation time course [57].

In this work, we found that incorporation into the Kv7.1 channel tetramer of an inactivating subunit (L273F) incrementally stabilizes the inactivated state and contributes to inactivation gating by progressively increasing the extent of inactivation and slowing down the recovery kinetics from inactivation. The incremental number of mutant subunits of the Kv7.1 tetramer does not significantly affect the time constants of the inactivation kinetics. Kv7.1 slow macroscopic inactivation is not compatible with the N-type inactivation, where a single inactivation particle is necessary and sufficient to produce N-type inactivation of which the rate of recovery remains constant and is independent of the subunit composition. In contrast, each inactivating subunit incorporated into the Kv7.1 tetramer increasingly stabilizes the inactivated state. Thus, the more L273F subunits the Kv7.1 tetramer contains, the more time is needed to recover from inactivation. Kv7.1 slow macroscopic inactivation is not compatible either with a concerted cooperative interaction between the four subunits like in C-type inactivation [37,45,46]. For a concerted cooperative mechanism, which requires all four subunits to transit from the open to the inactivated state before the channel can inactivate, it is expected to obtain a similar ratio of steady-state to peak current. In contrast, we found that the ratio of steady-state to peak current decreases (or fractional inactivation increases) by incorporating an increasing number of inactivating subunits into Kv7.1 channels. An incremental number of subunits bearing the L273F mutation not only triggered a progressive increase in the extent of inactivation but also produced a gradual increase in the slope of the voltage-dependence of steady-state inactivation and a right-shift of the voltage-dependence of steady-state inactivation for the ConFFFF channel construct. The latter likely arises from the right-shift of the conductance-voltage relation of the Kv7.1 L273F mutant, compared to WT Kv7.1. A similar slow inactivation gating behavior is recurrently found in mutated residues of different regions of Kv7.1 [53,54]. Notably, mutations in the voltage sensor S4 (L233W and Q244W) produce a similar voltage-dependent slow inactivation [32], which suggests that the movement of the voltage sensor promotes slow inactivation. Kv7.1 slow inactivation gating likely inherits its voltage dependence from the activation gating process. Remarkably, we and others previously reported that Kv7.1 channels do not experience a late cooperative concerted opening transition but undergo sequential gating transitions leading to channel opening even before all VSD have moved [29–31,53,54]. The present work suggests that a similar behavior is adopted by Kv7.1 for slow inactivation gating, owing to the coupling between activation and slow inactivation.

## Materials and methods

### Molecular biology

Template DNA encoding human Kv7.1 was first cloned into the pGEM vector to generate the mutant subunits. The mutation L273F was introduced using standard PCR techniques with Pfu DNA polymerase. PCR amplified mutant products were verified by DNA sequencing. To perform the study, the wild-type (WT) Kv7.1 concatenated tetrameric construct (Con) was first built into the pGEM vector, where subunits D1, D2, D3 and D4 were connected by flexible linkers (8 glycines), each harboring unique restriction sites, EcoRI, XbaI and Hind III, respectively (Fig. 1A). The concatenated construct was confined by BamHI and BstEII restriction sites upstream and downstream, respectively. Using this cassette, each subunit could be removed and mutated separately by cut and paste with a pair of restriction enzymes. For example, the D1 subunit could be cut and paste using BamHI and EcoRI restriction enzymes. WT Con was subsequently used as a template for constructing the various mutant subunit combinations. The Con was then inserted into the pcDNA3 vector to allow eukaryotic expression in CHO and HEK 293 cells.

### Cell lines culture, cell culture, transfection and western blot

Chinese hamster ovary (CHO) cells were maintained in Dulbecco's modified Eagle's medium (DMEM) supplemented with 2 mM glutamine, 10% fetal calf serum and antibiotics, incubated at 370C in 5% C02. Cells were seeded on poly-D-lysine coated glass coverslips in a 24 multiwell plate, and transiently transfected with Transit LT1-Transfection Reagent (Mirus). pIRES-CD8 was co-transfected as a surface marker. For Western blotting, HEK 293 cells were grown as CHO cells and transfected using the calcium phosphate method. Cells were washed in PBS and lysed with a buffer containing 50 mM Tris-HCl [pH 7.5], 150mM NaCl, 0.1% Triton X-100, 1 mM EDTA, 1 mM PMSF and a protease inhibitor cocktail (1 hour at 40C, under rotation). Cell lysates were cleared by centrifugation (10,000 x g for 15min, 40C). Equal amounts of lysate proteins were resolved by 8% SDS-PAGE and blots were reacted using rabbit anti-Kv7.1 antibodies (Alomone Labs).

### Electrophysiology

Electrophysiological recordings were performed 40 hours after transfection, using the whole-cell configuration of the patch-clamp technique. Transfected cells were visualized using anti-CD8 antibody-coated beads. Data were sampled at 5 kHz and low pass filtered at 2 kHz (Axopatch 200A amplifier with pCLAMP9 software and a 4-pole Bessel low pass filter, Molecular Devices, USA). For voltage-clamp measurements the patch pipettes were pulled from borosilicate glass (Warner Instrument Corp, USA) with a resistance of 3–7 MΩ, and were filled with (in mM): 130 KCl, 5 Mg ATP, 5 EGTA, 10 HEPES, pH 7.3 (adjusted with KOH), and sucrose was added to adjust osmolarity to 290 mOsmol. The external solution contained (in mM): 140 NaCl, 4 KCl, 1.2 MgCl2, 1.8 CaCl2, 11 glucose, 5.5 HEPES, pH 7.3 (adjusted with NaOH), and sucrose was added to adjust osmolarity to 320 mOsmol.

### Data analyses

Data analysis was performed using the Clampfit program (pClamp 10.5; Axon Instruments), Microsoft Excel (Microsoft,Redmond, WA), and Prism 5.0 (GraphPad Software, Inc., San Diego, CA). Leak subtraction was performed off-line, using the Clampfit program of the pClamp 10.5 software. Chord conductance (G) was calculated by using the following equation: G = I/(V-Vrev), where I corresponds to the current amplitude measured at the peak current, and Vrev is the calculated reversal potential assumed to be −90 mV in CHO cells. G was estimated at various test voltages (V) and then normalized to a maximal conductance value, Gmax. Activation curves were fitted by one Boltzmann distribution: G/Gmax = 1/{1+exp[(V_50_-V)/s]}, where V50 is the voltage at which the current is half-activated and s is the slope factor. Fractional inactivation was measured by the formula: F = 1- R, where R is the ratio of the current amplitude at the end of the test pulse divided by that of the peak. To determine the steady-state inactivation of each L273F construct, the membrane potential was stepped to varying pre-pulse voltages from −70 mV to +50 mV in increments of 10 mV and to a subsequent test pulse at +50 mV. The different peak currents measured at the test pulse at +50 mV were divided by the largest peak current (at +50 mV) obtained from the −70 mV prepulse. These ratios of steady-state inactivation were plotted against the prepulse voltage and subsequently normalized. Steady-state inactivation curves (SS inact) were fitted by one Boltzmann distribution: I/Imax = 1/{1+exp[(V_50_-V)/s]}, where V_50_ is the voltage at which the current is half-inactivated and s is the slope factor. The recovery time from inactivation was determined from a −90 mV holding potential, by stepping the membrane voltage for 1.5 s to +50 mV to open the various Kv7.1 concatemer channels (first pulse), then repolarizing to −90 mV for various times to allow recovery from inactivation and stepping back to +50 mV for 0.5 s to reopen the channels (second pulse). The fractional recovery was calculated by the peak current of the second pulse divided by the peak of the first pulse. These ratios were plotted against the recovery times and the time constants of recovery from inactivation were calculated from an exponential fit (one phase association). All data were expressed as mean ± S.E.M. For electrophysiology, if not indicated otherwise, statistically significant differences were assessed by unpaired two-tailed Student's t test.
